# Scaling Invariance of Sports Sex Gap

**DOI:** 10.3389/fphys.2020.606769

**Published:** 2020-12-11

**Authors:** Lu Tang, Wenzheng Ding, Chengyi Liu

**Affiliations:** ^1^ Laboratory of Laser Sports Medicine, School of Sports Science, South China Normal University, Guangzhou, China; ^2^ School of Sports Science, South China Normal University, Guangzhou, China

**Keywords:** power law, scaling invariance, sport events, sports records, sex gap

## Abstract

The controversy over the evolution of sex gap in sports stems from the reported that women’s performance will 1 day overtake men’s in the journal *Nature*. After debate, the recent studies suggest that the sports sex gap has been stable for a long time, due to insurmountable physiological differences. To find a mathematical model that accurately describes this stable gap, we analyze the best annual records of men and women in 25 events from 1992 to 2017, and find that power-law relationship could be acted as the best choice, with an R-squares as high as 0.999 (*p* ≤ 0.001). Then, based on the power law model, we use the records of men in 2018 to predict the performance of women in that year and compare them with real records. The results show that the deviation rate of the predicted value is only about 2.08%. As a conclusion, it could be said that there is a constant sex gap in sports, and the records of men and women evolve in parallel. This finding could serve as another quantitative rule in biology.

## Introduction

Sport competitions are conducted to test the ability of individual athletes or teams, with the aim of quantifying and ranking their abilities. Since the athletic capacity of athletes is influenced by biology (MacArthur and North, 2005; Tanaka and Seals, 2008), training methods (Berthelot et al., 2015), environment (Haïda et al., 2013) and ecological rules, plus a touch of change, the annual best results of a given discipline will fluctuate over time. Although the athletic records are continuously breaking and re-forming, is the sex-based difference in sports also changing accordingly? For this problem, there have been many studies that investigate the past world records of different sport events to analyze the change of sex gap. Over the past 2 decades, the conclusions related to this topic have aroused fierce controversy. Finally, the debate focuses on the changing trends of sports sex gap.

A number of studies considered that men may continue to improve on some events, and believed that the sex gap may slightly increase before reaching full stability (Holden, 2004; Seiler et al., 2007; Lepers, 2008). Several previous studies used a linear model to predict that the progress of physical performance for men and women (Dyer, 1986; Whipp, 1992; Tatem, 2004). One of such studies used the linear regression to fit human performance in sprint in the 20-second century, and extrapolated the fitted line to subsequent years (Tatem, 2004). And this study predicted that the women’s 100 m race could be won in a time of 10.57 ± 0.232 s and the men’s event in 9.73 ± 0.144 s in 2008 Olympiad, and that women will surpass men for the first time in 2056 Olympiad. However, other studies had cast doubt on the illogical predictions obtained by using simple linear regression analysis (Reinboud, 2004; Cheuvront et al., 2005; Chang and Baek, 2011). For example, Cheuvront et al. (2005) compared historical world record running performances for men and women to include sprinting events, and found that including world record-setting running performances for women before and after 1985 results in a non-linear data fit. After analyzing sex differences from the physiology perspective, they believed that men possess a larger aerobic capacity and greater muscle strength, and the sex gap in sports is unlikely to narrow naturally.

More recently, to measure the evolution of sports sex gap, Thibault et al. (2010) compared the improvement of male and female world records and 10 best performances between 1896 and 2007, and they result shown that the sex gap in Olympic sport performance had been stable since 1983. Since then, Thibault’s views seem to have become the mainstream voice. In this paper, in addition to verify the correctness of Thibault’s conclusion, another purpose is to find a mathematical model to describe the phenomenon of stable sex gap in sports.

The fractal model describes a self-similar pattern in different space or time scale, also known as scale invariance (West, 2010). Fractal physiology is widely used to study how fractal temporal structures in physiological fluctuations generated by complex physiological networks (Ivanov et al., 1996; Bernaola-Galván et al., 2001; Hausdorff et al., 2001; Wang et al., 2005; Tolkunov et al., 2010). Some groups studied the scale-invariant properties of heartbeat sequences. It had been found that the observed multifractality was related to nonlinear features of the healthy heartbeat dynamics (Peng et al., 1993; Thurner et al., 1998; Ivanov et al., 1999, 2001). One of such studies had shown a clear loss of multifractality for congestive heart failure (Ivanov et al., 1999). In addition, the observation of scaling behavior also extended to other physiological time series, such as gait rhythm (Hausdorff et al., 2001), respiratory rhythms (Peng et al., 2002), wrist activity (Hu et al., 2004), and foot pressure (Gilfriche et al., 2018). The scaling laws strongly depend upon the state of the underlying physiologic control system (Ivanov et al., 2001), so the abnormal scaling behavior of the above physiological signals can be used as an important diagnostic approach for related diseases.

Scale invariance describes phenomena that are not associated with a particular or characteristic scale of length, energy, or other variables, and is mathematically equivalent to power law behavior. Many studies have used power law to analyze the law of sport (Katz and Katz, 1994; Sylvan Katz and Katz, 1999; Vincenzo and Sandra, 2001; Yamamoto, 2009; Fernández-Revelles and García Mármol, 2019). Among them, Katz’s research shown that the performance of male and female superior athletes exhibits a fractal relationship between world record running and swimming times and the distance of the even, and an exceptionally good linear fit (*R* ^2^ ≥ 0.999) was observed in the log-log plot (Katz and Katz, 1994). In the paper, we collect the annual best results of men and women in 25 events from 1992 to 2018, and will use power-law relationship to reveal the evolution law of sports sex gap.

## Data Collection and Methods

### Data Collection

The data collected in this study are the annual world’s records for men and women from the racing and jumping sports, including running, marathon, swimming, high jump, long jump, triple jump, and hurdles for a total of 25 events. Considering the low participation of women before the 1980s (Dyer, 1986; Thibault et al., 2010) and the use of drugs to enhance performance in sports had certainly occurred during the 1970s and 1980s, we only collected the annual records after 1992 (Holden, 2004). All data were obtained from the “International Association of Athletics Federations (IAAF) World Championships Doha 2019 Statistics Handbook” (Butler, 2019), the websites of the IAAF^1^ and Fédération Internationale de Natation Association (FINA; www.fina.org). A total of about 1,300 athletes were considered in the study.

### Data Processing and Analysis

The data processing starts by organizing the downloaded data in a Microsoft Excel 2010 document (for data set, see Supplementary Table S1). To maintain consistency with the chronological records, such as running, swimming and hurdles, the records of high jump, long jump, and triple jump were rendered by the reciprocal transformation. In this study, it is used the power law equation (Harte, 1999; Sylvan Katz and Katz, 1999; Hu et al., 2004; Fernández-Revelles and García Mármol, 2019), namely *y* = *ax^l^*, to describe sex differences in sports. We define variables *x* and *y* to represent the male and female sports records of an event, respectively. The statistical software SPSS is used (Version 20, IBM) to organize the data in variables. Before visualizing the functional relationship between *x* and *y*, we first take log of them, then perform a linear fit, and finally draw a log-log plot. The effect of fitting is quantitatively described by the coefficient of determination. Each point in the plot corresponds to the male and female records of an event in a certain year. So, we will get a scatter plot with 650 points. Ideally, if the power law model is satisfied between *x* and *y*, all points will fall on a straight line, and the function of the line is log *y* = *l* log *x* + log *a*. The slope and intercept of the line will be determined by linear fitting, so that the parameters *l* and *a* in the power law equation can be calculated.

The above process is similar to the previous study in which the power law model is applied to athletic performance analysis (Sylvan Katz and Katz, 1999; Vincenzo and Sandra, 2001).

## Results

### Evolution of Sports Sex Gap

**Figure 1:**
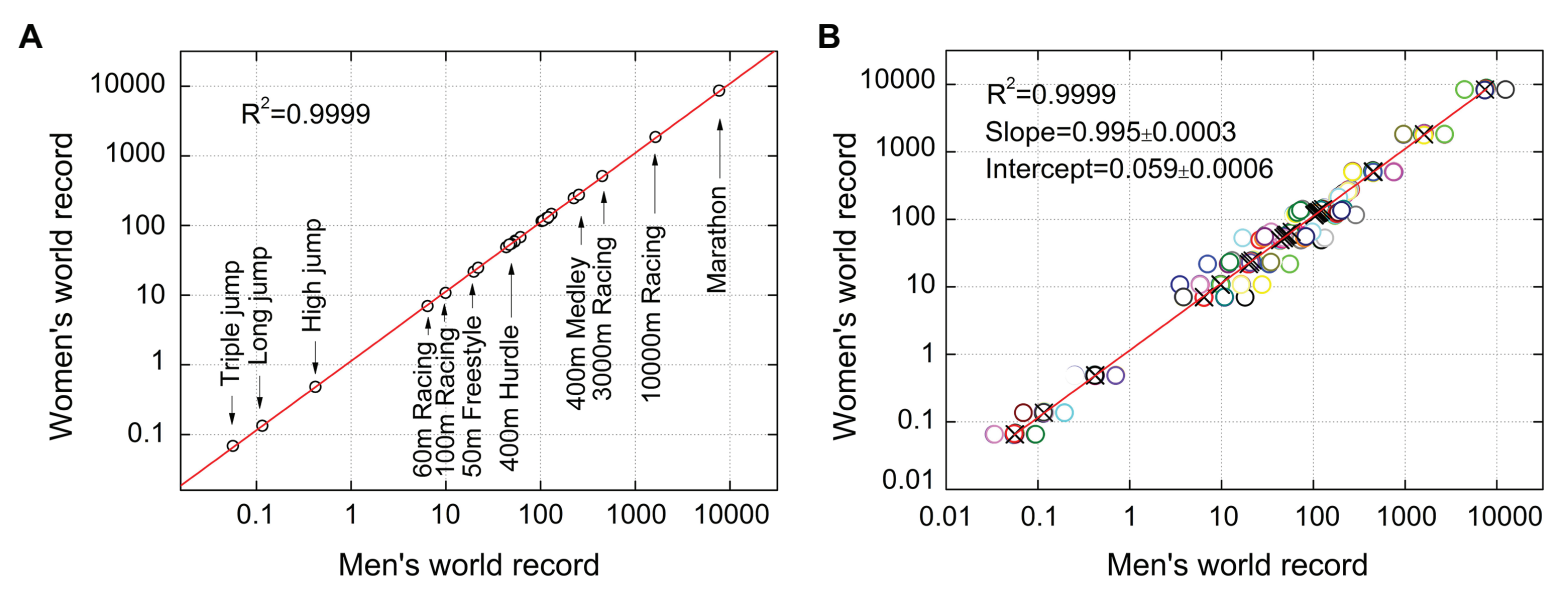
The power-law relationship between male and female records in 1992 **(A)** and 1992 to 2017 **(B)**, respectively.

Following the above procedure, we obtain the double-logarithmic coordinate plots describing the functional relationship between male and female records. To facilitate the identification of each sample point on the plot, firstly, we only analyze the male and female records of 25 events in 1992. As shown in Figure 1A, each circle in the figure represents an event (some have been marked). The results of linear regression analysis show that all circles fall on this fitting line, and exhibit an exceptionally good fitting degree (*R* ^2^ ≥ 0.999, *p* ≤ 0.001). Then, to quantify the parameters of the regression line, we present the male and female records of all events from 1992 to 2017 in a coordinate plot with 650 circles. Since many circles are overlapped together, so we plot with overlapped points offset, and the centers of the circles are shown by crosses. The result of linear fitting is shown in Figure 1B. The slope and intercept of regression line are 0.995 and 0.059, respectively. The SDs of both parameters are in the order of 10e^−4^. The coefficient of determination *R* ^2^ for linear regression is 0.9999 (*p* ≤ 0.001). This result shows that the fitness of the power law model is much higher than that of other reported mathematical models (Cheuvront et al., 2005; Thibault et al., 2010). In addition, according to the power law equation log *y* = *l* log *x* + log *a*, we can calculate the parameters *l* = 0.995 and *a* = 1.146.

### Stable Ratio Between Male and Female Records

**Figure 2:**
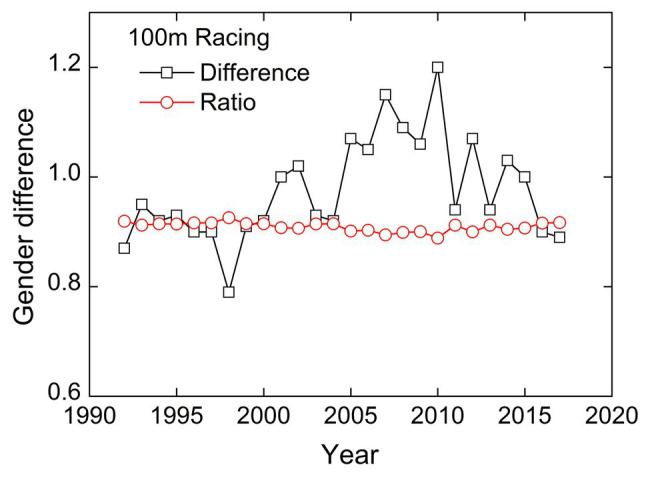
The fluctuation of sports sex gap in 100 m racing. The coefficient of variation (CV) of the ratio and difference is 0.922 and 9.466%, respectively.

Beyond that, it is worth noting that the slope of regression line is approximately equal to 1, that is, the first-order form of the power-law relationship. The power law equation is rewritten as *y* = *ax*, which shows that *a* is the ratio of male and female records. So, maybe we can describe sports sex differences in a simpler form. Under first-order approximation, the ratio fluctuation of sex difference in 100 m racing is shown in Figure 2. Meanwhile, as a comparison, the difference fluctuation of the records is also given. We use the coefficient of variation (CV) to quantify the stability of the two different description methods. The calculated CV of the ratio and difference between male and female records are 0.922 and 9.466% respectively, and they have one order of magnitude difference. Therefore, compared to the difference, the ratio can correctly evaluate the law that sex differences tend to stabilize. This conclusion is consistent with research of Thibault et al. (2010) to describe the invariance of sex gap using relative differences. Difference and ratio are two forms to describe the difference of two certain values, and the former is more commonly used. In fact, the paper published in the journal of *Nature* extrapolated that women will soon outrun men based on the intersection of the fitting lines of their performances (Tatem, 2004), so they are essentially measuring sports sex gap in the form of difference.

### Forecasting World Records by Scaling Laws

Prior to this, we find that there is a power-law relationship between men’s and women’s sports records. Based on the data from 1992 to 2017, the parameters *l* and *a* in the power law model are calculated. Here, we will forecast the women’s performance in 2018 by using the men’s performance of that year, on the basis of the two parameters. The true and forecasting world records for 25 events are shown in Table 1. The highest deviation between the predicted value and the real value is 3.92% for running events and 2.03% for swimming events. Among them, the deviation for the 800 m middle-distance running events is the lowest, only 0.02%. In addition, we notice that although the relative deviations are only about 3.24% (23 ms) and 2.19% (24 ms) for 60 and 100 m running, respectively, this variation might be considered as a significant in this type of events. Considering the characteristics of sprint events, environment, especially wind speed, is the key factor affecting the performance (Janjic et al., 2017). For example, a typical trailing wind of 2 ms^−1^ will confer benefits (0.5–0.8%) on the three sprint events (Hollings et al., 2012). To this end, we review the wind speed information of men’s and women’s 100 m competition in 2018. It is found that the records of 100 m for men and women in 2018 are obtained at wind speeds of −0.3 and +1.5 m/s, respectively. Therefore, we believe that this is the main factor that leads to the actual record to be 24 ms less than the predicted value.

**Table 1:** Comparison of predicted and actual values of female records in the 2018 sports events.

Events (unit)	Real records		Predicted value of female records	Relative deviation (%)
	Male	Female		
60 m running (s)	6.34	6.97	7.20	3.24
100 m running (s)	9.79	10.85	11.09	2.19
200 m running (s)	19.65	21.89	22.18	1.31
400 m running (s)	43.61	48.97	49.02	0. 11
800 m running (s)	102.05	114.25	114.23	0.02
3,000 m running (s)	448	507.5	497.76	1.92
10,000 m running (s)	1633.01	1841.85	1802.70	2.13
Marathon (s)	7260.39	8280.11	7955.27	3.92
100 m backstroke (s)	48.88	55.81	54.91	1.60
200 m backstroke (s)	107.02	119.94	119.76	0.15
100 m breaststroke (s)	56.01	62.74	62.88	0.23
200 m breaststroke (s)	120.16	135.62	134.39	0.91
100 m butterfly (s)	49.22	54.84	55.29	0.83
200 m butterfly (s)	108.24	121.6	121.12	0.39
50 m freestyle (s)	20.33	23.19	22.94	1.08
100 m freestyle (s)	44.95	51.01	50.52	0.96
200 m freestyle (s)	101.15	111.38	113.22	1.66
400 m freestyle (s)	214.01	233.92	238.66	2.03
200 m medley (s)	111.01	123.25	124.20	0.77
400 m Medley (s)	236.43	261.4	263.53	0.82
4 × 100 m freestyle (s)	183.03	207.78	204.27	1.69
400 m hurdles (s)	46.98	52.75	52.79	0.08
1/Long jump (m^−1^)	0.12	0.14	0.13	5.95
1/High jump (m^−1^)	0.42	0.49	0.48	2.20
1/Triple jump(m^−1^)	0.06	0.07	0.06	3.15

## Discussion

In this study, a power-law relationship is presented between the sports performance of men and women for the collected data. In fact, since the human body involves complex interaction among many feedback systems (Bartsch et al., 2015); fractal distributions are found in a wide variety of physical and biological systems. There have been many previous studies investigating the power-law relationship between other sports variables. In one study, no less than five different fractals related to sports are mentioned (Katz and Katz, 1994), for example, the distance of the event, the reciprocal of the total energy expended, and the ratio of aerobic and anaerobic energy consumption all have this relationship with the running or swimming time. The authors suggest that sports observers may regard fractal analysis as an important new instrument in their analytic tools. So, the findings of this article further support their view.

**Figure 3:**
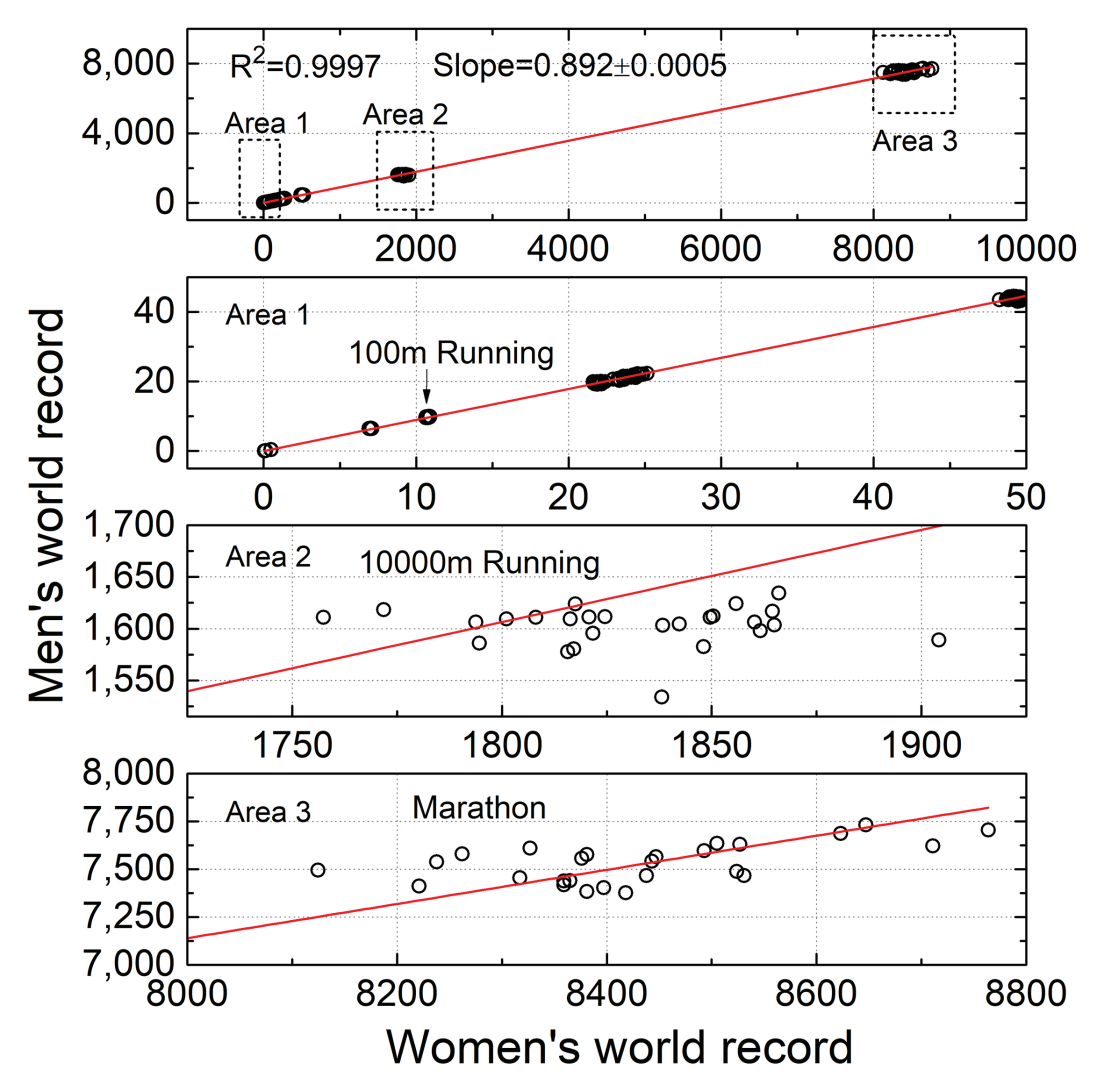
The linear correlation between male and female records in 1992 to 2017. The three sub-figures below correspond to the enlarged results of the three marked areas in the top figure.

It is worth noting that the results in Figure 2 show that the power law between men and women records, with an exponent close to one, which could also be interpreted as a simple linear proportionality between sex records. Therefore, we attempt to fit all collected the sports performance of men and women using a linear model with zero intercept (*y* = *ax*). As shown in Figure 3, each circle in the plot corresponds to the male and female records of an event in a certain year, with total of 650 circles. The fitting results show that *a* = 0.892 ± 0.0005 and *R* ^2^ = 0.9997 (*p* ≤ 0.001). We also zoom in on the local area of the figure (Area1, Area2, and Area3), and the three areas correspond to the data of 100 m running, 10,000 m running, and Marathon, respectively. As shown in the three sub-figures bellowed, it is easy to understand that the longer the running distance, the more circles deviate from the fitted line. Therefore, from the data collected so far, a linear model with zero intercept can be acted as a reasonable candidate for describing the evolution of sports sex gap. However, the predictive power of this approximate model will not be better than that of the power law model.

## Limitation and Practical Applications

Limitation to the current research study is acknowledged. Physiological differences between men and women, such as maximal oxygen uptake and muscle fiber cross-sectional area, are the main reasons for sex gap in sports (Cheuvront et al., 2005). If there is no any technological improvement specifically dedicated to one sex or the other, the gap will persist and become more and more stable as they reach their biological limits. However, the stability is also challenged by non-physiological factors, such as environmental conditions, sports participation, and doping. So, the accuracy of forecasting sports performance by power law model will be affected by these factors. Due to this limitation, a perturbation term or piecewise fitting can be selectively taken into account in the model in future.

As we know, the modern era of sport incorporates many technological elements, which promotes the steady improvements of athletes’ sports performance. But at the same time, the use of doping has become more and more hidden and sophisticated, which brings a lot of trouble to doping testing. Therefore, in order to maintain the fairness of the competition, international doping testing agencies should develop diversified testing methods to improve the technical level. In this paper, we find that the power-law relationship could be act as the best model for describing the evolution of sports sex gap. It indicates that the male and female sport records have the characteristics of parallel evolution, and one serious deviation from this law may be the result of using illegal performance-enhancing agents. Therefore, this discovery could provide an alternative method for performance forecasting and doping detection.

Finally, this article should emphasize that we are not in favor of treating female athletes differently. The data analysis in this paper only shows that there is a constant sex gap in sports that rely on physiological limits ability such as racing and jumping, while in other aesthetic sports such as synchronized swimming and rhythmic gymnastics, female athletes usually perform better than men. Furthermore, the level of sports performance does not mean who is better in cultural roles and social behaviors.

## Conclusion

Sex is one of the main determinants of sport performance, so the evolution of sex gap can be indirectly analyzed through the development of sports records over the years. In this letter, we collected the world records of men and women in speed and jumping events over the past 27 years and for the first time comprehensively analyzed the evolution of sports sex gap. The results show that the evolution of male and female records satisfies the power-law relationship with an exponent close to one. It implies that both from a physical and physiological point of view, the sport records of men and women have the characteristics of parallel evolution under the selection of training techniques, sport rules, and current socio-economic conditions.

Following the finding of various fractal distributions in sports statistics, this study observes that sports sex gap has the property of scale invariance. Perhaps, sports observers may regard fractal analysis as an important new instrument in their analytic tools.

## Data Availability Statement

The original contributions presented in the study are included in the article/Supplementary Material, further inquiries can be directed to the corresponding author/s.

## Author Contributions

LT and WD contributed to writing the original draft and data collection. WD contributed to the statistical analysis and visualizations, and revising and editing the manuscript. WD and CL conceived and supervised the project. CL supported the project. All authors contributed to the article and approved the submitted version.

### Conflict of Interest

The authors declare that the research was conducted in the absence of any commercial or financial relationships that could be construed as a potential conflict of interest.

## Footnotes

^1^www.iaaf.org
